# Fabrication of a Monolithic Implantable Neural Interface from Cubic Silicon Carbide

**DOI:** 10.3390/mi10070430

**Published:** 2019-06-29

**Authors:** Mohammad Beygi, John T. Bentley, Christopher L. Frewin, Cary A. Kuliasha, Arash Takshi, Evans K. Bernardin, Francesco La Via, Stephen E. Saddow

**Affiliations:** 1 Department of Electrical Engineering, University of South Florida, Tampa, FL 33620, USA; 2 Department of Medical Engineering, University of South Florida, Tampa, FL 33620, USA; 3 NeuroNexus Technologies, Inc., Ann Arbor, MI 48108, USA; 4 Department of Electrical and Computer Engineering, University of Florida, Gainesville, FL 32611, USA; 5 CNR Institute for Microelectronics and Microsystems, Catania, Sicily 95121, Italy

**Keywords:** neural interface, neural probe, neural implant, microelectrode array, MEA, SiC, 3C-SiC, doped SiC, n-type, p-type, amorphous SiC, epitaxial growth, electrochemical characterization

## Abstract

One of the main issues with micron-sized intracortical neural interfaces (INIs) is their long-term reliability, with one major factor stemming from the material failure caused by the heterogeneous integration of multiple materials used to realize the implant. Single crystalline cubic silicon carbide (3C-SiC) is a semiconductor material that has been long recognized for its mechanical robustness and chemical inertness. It has the benefit of demonstrated biocompatibility, which makes it a promising candidate for chronically-stable, implantable INIs. Here, we report on the fabrication and initial electrochemical characterization of a nearly monolithic, Michigan-style 3C-SiC microelectrode array (MEA) probe. The probe consists of a single 5 mm-long shank with 16 electrode sites. An ~8 µm-thick p-type 3C-SiC epilayer was grown on a silicon-on-insulator (SOI) wafer, which was followed by a ~2 µm-thick epilayer of heavily n-type (n^+^) 3C-SiC in order to form conductive traces and the electrode sites. Diodes formed between the p and n^+^ layers provided substrate isolation between the channels. A thin layer of amorphous silicon carbide (*a*-SiC) was deposited via plasma-enhanced chemical vapor deposition (PECVD) to insulate the surface of the probe from the external environment. Forming the probes on a SOI wafer supported the ease of probe removal from the handle wafer by simple immersion in HF, thus aiding in the manufacturability of the probes. Free-standing probes and planar single-ended test microelectrodes were fabricated from the same 3C-SiC epiwafers. Cyclic voltammetry (CV) and electrochemical impedance spectroscopy (EIS) were performed on test microelectrodes with an area of 491 µm^2^ in phosphate buffered saline (PBS) solution. The measurements showed an impedance magnitude of 165 kΩ ± 14.7 kΩ (mean ± standard deviation) at 1 kHz, anodic charge storage capacity (CSC) of 15.4 ± 1.46 mC/cm^2^, and a cathodic CSC of 15.2 ± 1.03 mC/cm^2^. Current-voltage tests were conducted to characterize the p-n diode, n-p-n junction isolation, and leakage currents. The turn-on voltage was determined to be on the order of ~1.4 V and the leakage current was less than 8 μA_rms_. This all-SiC neural probe realizes nearly monolithic integration of device components to provide a likely neurocompatible INI that should mitigate long-term reliability issues associated with chronic implantation.

## 1. Introduction

Implantable neural interfaces offer a method for external electronic devices to be connected to the central nervous system (CNS) in order to stimulate or record neurological signals, such as action potentials or multi-unit extracellular potentials, with the additional benefit of high spatial and temporal resolution. This interface forms a link for direct communication with the CNS through which the complex activities of neurons can be decoded to control active prosthetic devices or to stimulate one or more neural circuits to restore or enhance physiological functions [1]. 

Attempts to understand the electrophysiology of the nervous system started in the 17th century with stimulation of frog sciatic nerve [2]. Later in the 19th century, stainless steel wire electrode arrays were first implanted in the amygdala nuclei of monkeys and cats to investigate brain activity [3]. This was followed by the implantation of tungsten microelectrodes in the visual cortex of cats to investigate the behavior of individual cortical cells [4]. Study of the visual cortex, which requires a denser array of electrodes, drove a transition from metal wire electrode arrays to silicon-based three-dimensional microelectrode arrays (MEAs), such as the Utah array, which was introduced in the late 1980s [5,6]. This design minimized the electrode area and, as a result, allowed for higher spatial resolution during recording and stimulation of small populations of neurons, as well as utilized a reliable and repeatable manufacturing process. The high density of electrode sites, ability to individually address each electrode site, high-throughput fabrication, and compatibility with integrated circuit fabrication processes has made silicon an attractive material for high density, electrical neural interface applications. 

A milestone in the development of silicon-based implantable intracortical neural interfaces (INIs) was the Michigan probe, introduced in 1970 [7], which employed multiple electrode sites on a single shank for chronic intracortical stimulation of, or recording from, single neurons [8]. Nevertheless, the occurrence of mechanical, material, and biological failures, both acute and chronic [9], has been a major factor in the questionability of silicon- and metallic-based micro-INIs for human utilization. Mechanical failure, in the form of lead or connector breakage, material degradation, or insulation delamination, and biological failures, such as bleeding, cell death, meningitis, gliosis, and fibrotic encapsulation and extrusion, have been reported elsewhere [10]. In one report, collected from an evaluation of 78 silicon-based intracortical MEAs chronically implanted in rhesus macaques, nearly half of the chronic failures happened within the first year [11]. The majority of those chronic failures (53%) were reported as biological failure caused by meningeal encapsulation and extrusion from the tissue. These results indicate the importance of a mechanically and chemically robust INI that offers better compatibility with the CNS to provide long-term recording and stimulation capabilities. 

In recent years, researchers developing neural implants have turned their focus to flexible materials and designs to develop tissue-like INIs that address both mechanical and form factor compatibility. One implementation is an ultra-flexible, polymer-based probe in which a metal layer is sandwiched between two layers of SU-8 polymer [12]. Although this polymer-metal probe, and other similar designs [13,14], have shown a reduced immune response and were able to record action potentials and stimulate neurons, difficulty in the fabrication of these polymer-based devices, insertion of flexible polymer probes into the brain, and oxidation still remain fundamental issues [15]. Another method used to enhance the biocompatibility of neural probes are coatings that alter surface chemistry to provide hemostatic or immunomodulatory support [16]. In one example [17], a L1 protein coating was used to reduce microglial surface coverage. However, the surface coatings lose effectiveness over time leading to increased impedance and reduction in the recorded signals, and, in some cases, the mechanisms through which modulation of neurodegeneration and the corrosive behavior of encapsulating cells occurred was unclear [18].

For an INI to stimulate and record neural signals reliably over many years, both choice of material and their homogeneity must be carefully taken into consideration. Crystalline silicon carbide (SiC) is a semiconductor with a short bond length that gives it high physical resilience and chemical inertness. One of the important properties of SiC is that it displays polymorphism, which results in numerous single-crystal forms with the principal being hexagonal (i.e., 4H- and 6H-SiC) and cubic (i.e., 3C-SiC). SiC has been used in both the high-power electronics and MEMS industries [19,20]. It has also demonstrated a high degree of biological tolerance in vitro [21,22,23,24]. In addition, amorphous SiC (*a*-SiC), which provides excellent electrical insulation, has also shown good compatibility with neural cells [24,25,26] and has previously been used in the fabrication of several types of MEAs [27,28,29,30,31,32]. The properties of crystalline and amorphous SiC, and the results of previous studies, indicate that SiC can address the interrelated issues of INI biocompatibility and long-term reliability.

In our previous work, we reported the fabrication and characterization of nearly monolithic MEAs made from 4H-SiC, a hexagonal polytype of crystalline SiC, with *a*-SiC insulation [33]. However, the manufacture of these devices, as well as their release from the bulk SiC wafer, made these devices difficult to fabricate and costly. Here we report on the design and fabrication of a Michigan-style SiC neural probe on a silicon-on-insulator (SOI) wafer for ease of manufacture. The probe is composed of 3C-SiC, which was epitaxially grown on a SOI wafer. A heavily doped n-type (n^+^) 3C-SiC film was grown on a moderately doped p-type SiC layer, forming a p-n junction. The n^+^ layer was used to form the traces and electrode sites, eliminating the need for metallic conductive traces and metallic electrode sites that are in direct contact with the CNS tissue. The p-n junction structure provides substrate isolation between the conductive traces. A thin film of *a*-SiC was deposited via plasma-enhanced chemical vapor deposition (PECVD) on the probe to provide insulation from the external environment. The oxide buried in the SOI wafer served as a sacrificial layer, allowing the SiC probe to be released from the wafer with a selective wet etch process. This new fabrication approach, based on an all-SiC probe design, eliminates residual stresses typically found in similar devices consisting of stacks of heterogeneous films. It is expected that this approach will enhance the long-term material stability of implantable neural probes in the CNS, therefore increasing device reliability over many years. However, now that the manufacture of the probes has been demonstrated, follow-on studies in laboratory animals is required to support this hypothesis and are in the planning stages.

## 2. Materials and Methods 

The all-SiC neural probe was developed using variations of standard silicon semiconductor micromachining processes. This started with epitaxial growth of a 3C-SiC film on a SOI wafer [20], followed by patterning of the 3C-SiC epitaxial films via thin film contact photolithography techniques. This was followed by the subsequent etching of features using a deep-reactive ion etcher (DRIE), deposition of a conformal *a*-SiC film via PECVD, and a final probe definition etch through the buried oxide layer via a DRIE process. The final step was the release of the finished device from the substrate SOI wafer by wet etching the buried oxide layer. The thickness of the doped epitaxial films was measured using cross-section scanning electron microscopy (SEM) and the composition was verified through energy-dispersive X-ray spectroscopy (EDS). No S-peak was observed in the EDS spectrum, indicating that the device surface was free of chemical residue from the etch processes. A commercial connector (Nano Strip, Omnetics Connector Corporation, Minneapolis, MN, USA) was used to make the electrical connections to the probe. Planar single-ended test microelectrodes were fabricated from the same epiwafer material as the implants for ease of electrical testing. Cyclic voltammetry (CV) and electrochemical impedance spectroscopy (EIS) in a phosphate buffered saline (PBS) solution, as well as p-n junction isolation and leakage current tests, were conducted on the test microelectrodes to electrically characterize the fabricated probes.

### 2.1. Epitaxial Growth of 3C-SiC on SOI

A 100 mm diameter SOI ((100) Si-oriented) wafer, with an ~26 μm silicon film on top of an ~2 μm buried thermal oxide layer, was used for fabrication of the all-SiC neural probes reported here. The growth process started with epitaxial growth of an ~8 μm p-type 3C-SiC film on the SOI wafer, followed by an ~2 μm heavily n-type (n^+^) film, using a hot-wall reactor (LPE Epitaxial Technology, Baranzate, Italy) [34]. Heavy doping of semiconductors results in semi-metallic performance, which is the case for 3C-SiC. For this to be achieved, a n^+^ doping density of ~10^19^ dopants/cm^3^ is required. Hydrogen (H_2_) was used as a carrier gas [19], ethylene (C_2_H_4_) as the carbon precursor, and trichlorosilane (SiHCl_3_) as the silicon precursor gas. The epitaxial growth temperature was set to ~1370 °C with a process pressure of ~75 Torr. The C:Si ratio was kept between 0.8 and 1.2 throughout the epitaxial growth process. Aluminum and nitrogen were the p and n dopants, respectively, and were introduced during the epitaxial growth process [35,36]. The doping level of the top n^+^ 3C-SiC film was measured with a LEI 2017b Mercury (Hg) Probe (Lehighton Electronics, Inc., Lehighton, PA, USA) [33].

### 2.2. Fabrication of All-SiC Neural Probe

**Figure 1:**
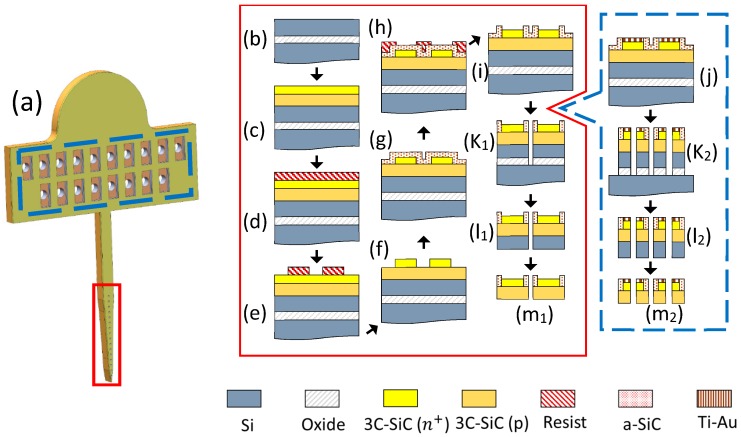
The all-SiC fabrication process flow. (**a**) A rendering of the Michigan-style 3C-SiC probe. The process flow inside the red rectangle shows the cross-section at the electrode sites while the blue rectangle provides the cross-section at the contact pads on the tab. (**b**) Starting SOI wafer, (**c**) ~8 µm of p-type 3C-SiC was grown on top, followed by ~2 µm of heavily n-type (n^+^) 3C-SiC. (**d**) The wafer was coated with photoresist and (**e**) patterned via photolithography. (**f**) DRIE process was used to form the conductive n^+^ mesas and (**g**) a thin *a*-SiC insulating layer was deposited on top via PECVD. (**h**) Photoresist was then patterned with photolithography and (**i**) the *a*-SiC was etched to form windows for the electrode sites using a RIE process. (**j**) After the *a*-SiC windows were opened, a layer of titanium, followed by gold, was deposited on the contact pads and thermally annealed. A deep DRIE etch through both epi layers and the oxide was performed to (k_1_) define the probes and (k_2_) form through-holes in the contact pads. (l_1_, l_2_) The oxide layer was etched in HF (49%) to release the probes. (m_1_, m_2_) Back-thinning via DRIE was performed to remove the residual silicon from the SOI device layer.

The fabrication process sequence is shown in Figure 1. First, the epiwafer with the SiC films was cleaned in a solvent and then a RCA bath. It was then dipped in hydrofluoric acid (HF, 49%, J. T. Baker, Inc., Phillipsburg, NJ, USA) diluted in water (50:1) to remove any oxide that may have formed on top of the epitaxial 3C-SiC layer, followed by a DI water rinse and N_2_ dry. Next, the wafer surface was functionalized with HMDS (Hexamethyldisilazane; Microchemicals GmBH, Ulm, Germany) and a 15–18 μm layer of AZ 12XT-20PL positive photoresist (Microchemicals GmBH) was spun on top at 1500 rpm. After a soft-bake at 110 °C for 3 min, the photoresist was patterned by UV exposure (110 mJ/cm^2^) with a Quintel Mask Aligner and then baked at 90 °C for 1 min. The wafers were re-hydrated at ambient condition for 2 h and then developed with AZ300 developer (Microchemicals GmBH). The patterned photoresist was thick enough to allow for a ~3 μm deep etch of the epitaxial film using an Adixen AMS 100 DRIE. This process used oxygen (O_2_) at 10 sccm and sulfur hexafluoride (SF_6_) at 90 sccm. The pressure inside the chamber was set to 5.7 mTorr and the sample holder temperature was set to −20 °C. The sample holder power was kept at 550 W, while the source RF power was 1800 W. This process formed the traces and electrode sites on the probes. 

A ~250 nm layer of *a*-SiC was deposited on the sample using PECVD (Unaxis 790, PlasmaTherm, Saint Petersburg, FL, USA). Methane (CH_4_) and silane (SiH_4_, 5% in He) were used as reactive gases to produce the *a*-SiC with flow rates of 200 sccm and 300 sccm, respectively. Helium (He), with a flow rate of 700 sccm, was used as the carrier gas. The RF power was set to 200 W, substrate temperature to 300 °C, and pressure to 1100 mTorr [37,38]. Following photoresist patterning using AZ 15nXT-450 CPS negative photoresist (Microchemicals GmbH), a reactive ion etch (RIE; PlasmaTherm) was run for 210 s to open windows in the *a*-SiC film for the electrode sites. Tetrafluoromethane (CF_4_) and O_2_, at 37 sccm and 13 sccm respectively, were used as the process gases. The power was set to 200 W and the chamber pressure to 50 mTorr. In order to package the probes for electrical testing, metal bonding pads were formed on one end of the traces (for the implants this is located on a tab that would reside outside the skull of the animal during in vivo testing). A 20 nm titanium (Ti) film, followed by a 200 nm gold (Au) film, was deposited without breaking vacuum in an electron beam evaporator and patterned using a lift-off process. Thermal annealing was performed to create an ohmic contact at the interface between the semiconductor and metal in a rapid thermal processor at 650 °C for 10 min [39]. This process sequence formed the contact pads for the commercial connector, which was used to connect the electrodes to external electronics.

The last step of the fabrication process was probe release. The same DRIE etch recipe that was used for formation of the traces was employed in an etch-through process to define the probes, except that the duration was increased to 15 minutes in order to ensure complete through etch of the 3C-SiC epitaxial films and top silicon layer. A scrap piece of the epiwafer was cleaved and cross-section SEM was used to determine the 3C-SiC epilayer and Si device layer thickness so that proper etch depth and mask thickness were selected. After removing the photoresist, the etch depth was measured using a contact profilometer (Dektak 150, Veeco, Plainview, NY, USA). The probes were released via wet etch of the sacrificial oxide layer with HF (49%). Then they were carefully removed from the HF solution, rinsed with DI water, and dried with N_2_. To remove the backside silicon, the probes were adhered upside-down to a Si handle wafer with ~1 μm thermally grown oxide on top using a thin photoresist layer and placed in the DRIE. The residual Si was removed using the same DRIE recipe used for the definition of the electrodes and traces.

### 2.3. P-N Junction Isolation and Leakage Evaluation

Since p-n junctions are formed between the n^+^ and p epitaxial films, back-to-back diodes are present between adjacent traces, which provides isolation. This isolation was evaluated by measuring the forward and reverse blocking voltages of test structures consisting of p-n diodes and n-p-n junctions formed between adjacent traces that were built on the 3C-SiC wafer. A Keithley 2400 SourceMeter (Tektronix, Inc., Beaverton, OR, USA) was used to generate current-voltage (I-V) plots for adjacent traces to observe these voltages. The voltage was increased from −10 V to +10 V at a rate of 0.1 V/s for the diodes and n-p-n junctions, and the observed currents recorded. The forward voltage was estimated using a semi-logarithmic current scale I-V plot [40]. The breakdown voltage occurs when the current rapidly increases during application of negative voltage. The root mean square (rms) of the current amplitude between breakdown and forward potentials for the diodes was defined as reverse leakage current [33]. The threshold current for defining the breakdown voltages was 10 µA.

### 2.4. Electrochemical Characterization of All-SiC Probes 

Electrochemical characterization of the 3C-SiC electrodes was performed via CV and EIS evaluation. A three-electrode setup was used with a potentiostat (VersaSTAT 4, AMETEK, Inc., Berwyn, PA, USA) to adjust the voltage between the working and counter electrodes in the presence of a reference electrode. CV provided information on the charge transfer properties of the electrode-electrolyte interface and on the presence of electrochemical reactions and their reversibility. Potential limits of −600 mV and +800 mV, which is the electrochemical window for platinum (Pt), were chosen for CV because this allowed for direct comparison of our measurements with previously published results [27,32,41,42]. EIS provided complex impedance measurements (both magnitude and phase) at frequencies of interest, which were used to evaluate the performance of the n^+^ 3C-SiC conductor traces and electrodes.

Planar test microelectrodes fabricated alongside the neural probes on the same wafer were used for CV and EIS measurements [33,37]. The measurements were performed at room temperature in PBS with a pH of 7.40 ± 0.01, which was adjusted with hydrochloric acid (HCl). The PBS was composed of 137 mMol NaCl, 2.7 mMol KCl, and 10 mMol Na_2_HPO_4_. The gas levels in the PBS were ambient and no bubbling was done. The counter electrode was Pt and the reference electrode was Ag|AgCl. EIS measurements were performed from 0.1 Hz to 1 MHz with a rms voltage of 10 mV. The current was recorded 12 times per decade and three repetitions were averaged. CV measurement was initiated from open circuit potential, swept to −600 mV, and increased to +800 mV at a rate of 50 mV/s. This cycle was repeated three times and results were averaged. Charge values were calculated from the CV I-V curve via numerical integration with the trapezoidal method, trapz, in MATLAB (MathWorks, Natick, MA, USA).

## 3. Results

### 3.1. Epitaxial 3C-SiC Films

**Figure 2:**
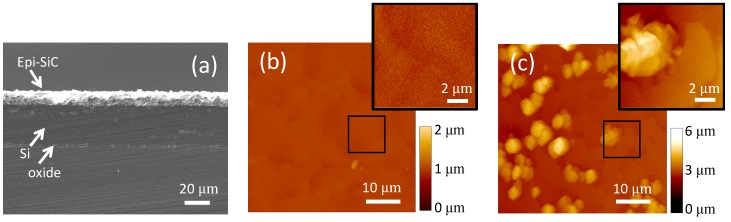
Analysis of epitaxial SiC results. (**a**) Cross-section SEM micrograph of the 3C-SiC epi films on SOI. (**b**) AFM image (tapping mode) of the 3C-SiC epiwafer specular region on the wafer periphery that shows typical 3C-SiC surface morphology (mean roughness of ~21 nm). (**c**) AFM image (tapping mode) of the rough surface of the same epiwafer (center) (mean roughness of ~244 nm). The devices were fabricated from the center of the wafer.

A cross-sectional SEM view of the wafer, which allows for accurate estimation of film thickness (n^+^-, p-SiC, Si device film, and buried oxide), is shown in Figure 2a. This figure highlights various layers and the approximate thickness of each layer on the wafer used for the fabrication. The two epitaxial 3C-SiC films were measured, and their combined thickness determined to be ~10 μm. The SOI Si device layer (~26 µm), as well as the thin (~2 µm) buried oxide layer are also visible in this figure. The epitaxial n^+^ film in the center of the wafer was quite rough with a mean surface roughness of ~244 nm and smoother near the wafer edge with a mean surface roughness of ~21 nm. Figure 2b shows surface morphology of the smooth n^+^ layer, which was taken using a DI AFM (Dimension 3100). Although rough in the wafer center (Figure 2c), the surface roughness was low enough for thick layers of photoresist to properly cover the entire surface for the subsequent fabrication steps. However, this roughness would be expected to impact device electrical performance, particularly p-n diode leakage current.

### 3.2. Fabricated All-SiC Neural Probe

**Figure 3:**
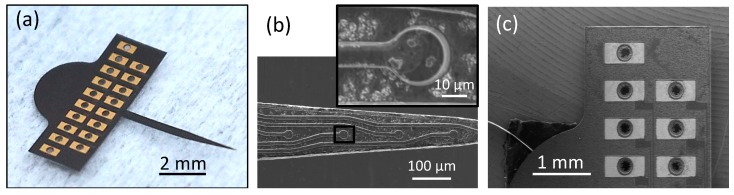
Physical characterization of the completed neural probe. (**a**) Optical image of a freestanding all-SiC probe after release. (**b**) SEM image of the shank tip showing four of the electrode sites and a magnified image of a single electrode site (inset). (**c**) SEM image of some of the metal contact pads with through holes. The shank is 5.1 mm long and the length of the tapered portion is 2.4 mm. The tab is 6.64 mm wide and 2.3 mm long, excluding the semi-circular top portion. The surface roughness of the electrode sites is shown in Figure 2c.

Epitaxial growth of single crystalline 3C-SiC with different types of doping enables realization of a nearly monolithic probe from homogeneous SiC material. The all-SiC probe is a Michigan-style, planar neural probe with 16 electrodes for recording and stimulating neurons. The connector tab has 18 metallic pads (approximately 0.8 mm by 0.4 mm) with through holes to which a commercial Omnetics connector is bonded. Two extra pads provide connections for the return and reference electrode wires. The diameter of the electrode sites is ~15 μm and width of the traces is ~10 μm. Figure 3 shows the optical and SEM micrographs of a free-standing probe.

The probe’s shank, which contains the traces and electrode sites, is shown in Figure 3b. This figure shows a scanning electron micrograph of the electrode sites, which have *a*-SiC windows on top to allow contact with the extracellular environment. The traces and electrode sites are mesas formed from the n^+^ 3C-SiC film. There are no metallic components on the shank, which is a homogeneous structure consisting entirely of SiC. The pads, which are shown in Figure 3c, contain titanium and gold layers in order to provide ohmic connections to external electronic devices via the Omnetics connector. However, since the metallic pads are not in direct contact with brain tissue, the issues regarding delamination of metallic parts and compatibility with CNS tissue are not a concern. 

### 3.3. Electrical and Electrochemical Characterization

The doping density of the top n^+^ 3C-SiC film was determined by measuring the capacitance voltage profile of the Schottky contact at 1 MHz and N_D_-N_A_ was estimated to be ~10^19^ cm^−3^. A similar measurement was also performed on the p-type epitaxial film exposed after DRIE processing and N_A_- N_D_ was estimated to be ~10^16^ cm^−3^. These measurements indicate the feasibility of p-n junction formation between two epi films and high electrical conductivity of the top semi-metallic n^+^ film that formed the traces and electrode sites. EIS was done to confirm this expectation.

**Figure 4:**
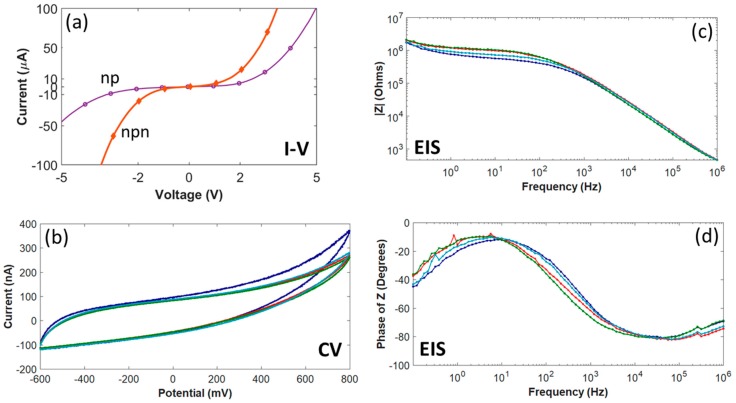
All-SiC p-n diode and n-p-n junction characterization and electrochemistry for four test microelectrodes with an area of 491 µm^2^. (**a**) I-V measured from a p-n diode and a n-p-n junction between adjacent traces fabricated on the same wafer used for probe fabrication. (**b**) The cyclic voltammetry curves swept between +800 mV and −600 mV with a scan rate of 50 mV/s. (**c**) EIS Impedance (Z) magnitude (~165 kΩ @1kHz) and (**d**) impedance phase angles. The curve for each microelectrode (b-d) is the average of three replicates.

As shown in Figure 4a, current-voltage (I-V) measurements on individual diode structures had a rectifying effect due to the diode formed between the n^+^- and p-type epitaxial films. In order to measure turn-on and breakdown voltages and the reverse leakage current, the I-V plot for four diodes on the same wafer was measured. The averaged turn-on voltage for these four diodes was determined to be ~1.4 V, with an average leakage current less than 8 µA_rms_. In addition, Figure 4a also contains a current-voltage curve, obtained from measurements on one of the IDEs, showing isolation between adjacent traces.

CV curves for four test microelectrodes of the same surface area (491 µm^2^) in 7.4 pH PBS are shown in Figure 4b. The upper (+800 mV) and lower (−600 mV) boundaries for the potential were based on the electrochemical window for Pt in water. The shape of the hysteresis cycle showed that the anodic and cathodic currents were charge balanced, with no indication of faradaic current resulting from oxidation or reduction reactions between +800 mV and −600 mV. However, the phase behavior of the electrode-electrolyte interface (Figure 4d) only supports a capacitive-dominant mechanism at higher frequencies (e.g., −61.2 ± 3.7° at 1 kHz), while at lower frequencies the phase indicates a faradaic current (e.g., −30.3 ± 4.9° at 100 Hz), which contrasts with earlier results from 4H-SiC microelectrodes [33]. The average anodic charge storage capacity (CSC) was 15.4 ± 1.46 mC/cm^2^ (mean ± standard deviation) and the cathodic CSC was 15.2 ± 1.03 mC/cm^2^. The average anodic charge per phase was 75.4 ± 5.06 nC and the average cathodic charge per phase was 74.8 ± 5.06 nC.

Figure 4c,d show the EIS results for the same four test microelectrodes. As expected, the impedance magnitude was found to increase with decreasing frequency. At a frequency of 1 kHz, the impedance was 165 ± 14.7 kΩ (mean ± standard deviation). The electrode-electrolyte interface was determined to be predominately capacitive, as indicated by the negative phase angles for higher frequencies (i.e., >1 kHz).

## 4. Discussion

A nearly monolithic SiC neural probe has been fabricated from epitaxial 3C-SiC films grown on SOI wafers. A combination of ethylene (C_2_H_4_) and trichlorosilane (SiHCl_3_) were used as precursor gasses in the epitaxial process. This produced a varying surface morphology with mean surface roughness of approximately ~21 nm (specular, edge region) to ~244 nm (rough, center region) [43]. It is possible this surface roughness contributed to complications in the fabrication process, such as with photolithographic patterning, and may have had an effect on the mechanical properties of the grown films to the detriment of probe function [44]. It is suspected that this contributed to the higher than desired leakage current (less than 8 μA_rms_). By optimizing various parameters in the epitaxial process, such as gas composition and flow rates, temperature, and pressure [35,43], the process can be improved to reduce this surface roughness. Additionally, post-processing steps, such as mechanical or chemomechanical polishing, can be added to further improve the surface morphology; particularly to reduce surface roughness [19].

A major issue with the previous 4H-SiC probes was the difficulty in releasing the probe [33]. Essentially, much of the 4H-SiC substrate would have to be removed, and there was no effective etch stop to prevent over-etching. In order to effectively solve this issue, we used SOI wafers to provide an effective release layer by the simple process of wet etching the oxide. However, the SOI wafer used here possessed a relatively thick layer of silicon that remained on the backside of the probes, which was removed later via back-thinning using DRIE. This thick silicon layer can cause residual stress, due to mismatches in the coefficients of thermal expansion and lattice parameters [45] at the interface between the SiC films and silicon, resulting in bowing or bending of the probes. The SOI wafer used in this work had an ~20 um thick top silicon layer and this may have been the cause of the bowing of the shank and some warping in the connector tab. The shank should be straight for a proper insertion trajectory into the neural tissue. Also, in order to maximize contact at the connector interface, the tab containing the contact pads should be as planar as possible. Using a SOI wafer with a thin silicon device layer may resolve this deformation problem and will be used in future all-SiC devices. 

Epitaxial 3C-SiC thin films are ceramic-like materials with, relative to neural tissue, a high elastic modulus, measured to be 424 ± 44 GPa using microsample tensile testing [46] and 433 ± 50 GPa using nanoindentation [47]. Defects can reduce the value the Young’s modulus of 3C-SiC [48] and doping may affect this value as well [49]. There is a trend towards utilizing softer materials, such as polymers, for implantable neural interfaces due to their potential to improve the interaction with neural tissue [50,51,52]. By decreasing the Young’s modulus of the neural probe closer to the values of neural tissues, the harmful shear and normal stress applied from the shank to the tissue should decrease. However, it is really device stiffness, which includes cross-sectional area, rather than just device modulus, that seems to matter the most [53]. Additionally, use of these softer materials introduce challenges with fabrication processes, scaling to higher channel-count systems, particularly with respect to interconnects, and can lead to insertion difficulties. Once implanted, these materials face challenges with material stability and device reliability [54]. The hard, chemically inert nature, and ease of micromachining with traditional silicon processes means SiC neural probes may suffer less from these limitations. Clearly, long-term in vivo studies in an animal model are needed to assess the performance of the all-SiC INI and are planned.

It has been demonstrated that once the implanted structure size is reduced to subcellular scale, i.e., less than ~10 µm, the foreign body response and associated neuron death is greatly reduced in a rat model [55,56]. With traditional silicon probes, reducing size increases the occurrence of probe fracture at high stress regions [57]. SiC is a much more robust material, with a reduced tendency to fracture at these desired smaller sizes, while maintaining the mechanical strength needed for proper penetration of neural tissue [24,27]. Therefore, SiC is an excellent material for developing a high electrode density neural interface, allowing for further reduction in size while greatly minimizing risk of fracture.

The heterogeneous composition of implanted neural interfaces that utilize metallic materials as electrode sites or conductive traces may increase the risk of delamination in chronic implantation, specifically, at regions under higher stress [57]. Delamination usually occurs at the interface between metal and semiconductor materials due to residual stress in the thin films. A homogeneous material composition can eliminate this residual stress, reducing the risk of delamination at the interfaces between different materials in the probe by eliminating them. 

The 3C-SiC is a wide-band-gap semiconductor with a high band energy of ~2.2 eV. This results in a higher turn-on voltage at the junction between n^+^- and p-type SiC. This higher turn-on voltage provides a wider voltage range to stimulate neurons while isolating individual channels via n-p-n junctions supporting simultaneous multichannel microstimulation and recording, as might be necessary for implementing a closed-loop system. The turn-on voltage for p-n junctions built from Si is ~0.7 V, which is low compared to ~1.4 V for SiC, and limits proper isolation via a n-p-n junction configuration. However, the higher leakage current in our all-SiC films may negatively affect the final device’s functionality. Surface roughness is known to be associated with the density of crystal defects, thus a higher defect density may cause higher leakage current [43]. It is believed that the high surface roughness in this work, an indication of poor crystallinity, in conjunction with a high number of defects, may be the cause of the observed high leakage current. For reference, in our 4H-SiC devices with specular surface morphology, the leakage current was nA versus µA reported here [33]. A lower surface roughness via an optimized epitaxial growth process would be expected to improve both the mechanical properties and the leakage current [58]. 

The EIS results revealed that the doped, semi-metallic 3C-SiC conductors have impedance values approaching those of metals commonly used in implantable microelectrodes, such as gold, platinum, or tungsten, as well as highly doped polysilicon [59,60]. The average impedance for a surface area of 491 µm^2^ was approximately 75% lower (165 kΩ vs. 675 kΩ at 1kHz) than previously reported for our 4H-SiC electrodes [33]. 

Both the charge balanced CV cycles and the negative phase angles from EIS measurements support a dominant capacitive charge transfer mechanism for 1 kHz and higher frequencies at the electrode-electrolyte interface, but faradaic currents may be present at lower frequencies. This differs from capacitive electrode materials like titanium nitride (TiN) [61], which has a phase closer to 90° at lower frequencies [62]. Compared to values previously reported for 4H-SiC, the charge values calculated from CV measurements reported here were approximately two orders of magnitude higher, with the average charge storage capacity (anodic: 15 mC/cm^2^ vs. 0.41 mC/cm^2^; cathodic: 15 mC/cm^2^ vs. 0.19 mC/cm^2^) and an average charge per phase (anodic: 75 nC vs. 2.0 nC; cathodic: 75 nC vs. 1.0 nC) using a Pt electrochemical window (−600 mV to +800 mV). It is possible that the greater surface roughness accounts for this large difference in electrochemical properties. It is also possible that there were more faradaic reactions at lower frequencies leading to more oxidation and reduction at the surface, which may be linked to defect sites in the SiC.

Current neural probe technology built from materials like silicon suffer from long-term reliability issues that reduces their lifetime considerably, resulting in loss of recording and microstimulation function when chronically implanted. This limits their use in medical applications for humans. Device-based modalities could become a more common alternative to pharmaceuticals for treatment of neurological trauma or disease if the issue of long-term reliability in implantable neural interfaces is properly addressed. After further refinement of the design and optimization of the material processing, the performance of the all-SiC neural probe will be evaluated with chronic *in vivo* experiments in rodent models to investigate its long-term safety and effectiveness in neural tissue. There is accumulating evidence [25,26,27,29,30,32,63] that SiC could be an appropriate material for the greatly needed implantable neural interface that functions for the lifetime of the recipient.

## 5. Conclusions

The fabrication and initial electrical characterization of an all-SiC neural probe is presented. The SiC neural probe was fabricated from p− and n^+^-type 3C-SiC epilayers grown on SOI wafers. First, a moderately p-type 3C-SiC film was grown on a SOI wafer, followed by a layer of n^+^-type 3C-SiC. The surface morphology of the top n^+^ epilayer was measured. Neural probes with sixteen traces, electrode sites, and other test structures were patterned on the 3C-SiC epilayers via MEMS microfabrication processes. Metallic traces were absent from the shank of the probe, and instead a semi-metallic n^+^ layer was formed into traces and electrode sites. A thin layer of *a*-SiC film was deposited on top of the epilayers to serve as an insulator. The probes were harvested using dissolution of the buried oxide layer in the SOI handle wafer to provide ease of manufacture. The backside silicon layer remaining after release of the probes was removed via back-thinning in a DRIE. Adjacent traces were electrically isolated through a n-p-n junction. After completion of device fabrication, the performance of the n-p-n junctions was evaluated through current-voltage measurements and the turn-on voltage was determined to be ~1.4 V. Electrical measurements showed satisfactory p-n junction performance, but leakage current needs to be improved via higher quality 3C-SiC epitaxial films. In addition, initial electrochemical characterization work with 491 µm^2^ surface area test microelectrodes demonstrated good impedance, charge storage capacity, and charge per phase values. These results support the feasibility of neural stimulation and recording with the fabricated all-SiC neural probe. However, further studies are necessary to demonstrate the acute recording and stimulation capability and chronic stability of the fabricated SiC neural probes, and, consequently, in vitro accelerated aging and in vivo studies in a rodent model are planned and will be reported in the future.
